# Enigmas of primary immunodeficiency and mycobacterial infection in our territory

**DOI:** 10.1186/1710-1492-10-S1-A30

**Published:** 2014-03-03

**Authors:** Roya Sherkat

**Affiliations:** 1 Acquired Immunodeficiency Research Center, Isfahan University of Medical Sciences, Isfahan, Iran

## Introduction

Defects of the immune system in Primary immunodeficient diseases (PIDs) predispose individuals to recurrent infections. Complex genetic components for susceptibility to mycobacterial disease have been suggested. Natural human immunity to the mycobacteria group, including Mycobacterium tuberculosis(MTB), Bacille Calmette-Guérin (BCG) or nontuberculous mycobacteria (NTM) relies on the functional IL-12/23-IFN-γ integrity of macrophages (monocyte/dendritic cell) connecting to T lymphocyte/NK cells [1]. Restricted defective molecules in the circuit and recently discovered CYBB responsible for autophagocytic vacuole and proteolysis have been identified in around 60% of patients with the Mendelian susceptibility to the mycobacterial disease (MSMD) phenotype [2].

Primary defects in oxidase activity in chronic granulomatous disease (CGD) lead to severe, life-threatening infections. The role of phagocytic respiratory burst in host defense against mycobacterium tuberculosis was controversial. Previous studied showed that the critical role at reactive oxidants is to serve as intracellular signals for activation of microbicidal enzymes, rather than excretions a microbicidal effect perse [3]. The role of phagocytic respiratory burst in host defense against M. TB is further supported by recent studies discovered immunological defects secondarily affecting phagocyte respiratory burst function and resulting in primary immunodeficiencies with varied phenotypes, including susceptibilities to pyogenic or mycobacterial infections [4].

The patients with severe PID’s like SCID have broader diverse infections susceptibility and mycobacterial infections as well, however, Common variable immunodeficiency (CVID) mostly characterized by a deficiency of immunoglobulins and recurrent sinopulmonary infections.

## Method

We overview the clinical rate of mycobacterial disease in our PID cases and evaluate the complex cases.

## Results

Two hundred PID cases were evaluate between 1996-2013 in our clinic, Among 5% of them which diagnosed as MSMD nearly all presented with mycobacterial infection. 8% diagnosed as CGD and interestingly 60% of them have been experienced mycobacterial disease sometimes in their life, as disseminated BCG or late onset complications of BCG including osteomyelitis or MTB once or more than one episode through their life. Also we have presented a CVID patient with disseminated TB and granulomatouse hepatitis, TB arthritis and peritonitis.

## Conclusion

PID cases Like CGD, MSMD or CVID which are living in area’s with high prevalence of mycobacterial infection could have quiet different presentations and the study of these complex cases has provided essential insights into the functioning of the immune system. Despite the conventional view we have confirmed that the generation of ROIS by phagocytic respiratory burst may play a role in the defense of the host against M. tuberculosis by clinical evidence.
